# Psychometric properties of the faces version of the Malay-modified child dental anxiety scale

**DOI:** 10.1186/s12903-015-0013-y

**Published:** 2015-03-10

**Authors:** Rashidah Esa, Noratikah Awang Hashim, Yuliana Ayob, Zamros Yuzadi Mohd Yusof

**Affiliations:** 1 Department of Community Oral Health & Clinical Prevention, Faculty of Dentistry, University of Malaya, 50603 Kuala Lumpur, Malaysia; 2 Community Oral Health Research Group, Faculty of Dentistry, University of Malaya, Kuala Lumpur, Malaysia; 3 Oral Health Division, Ministry of Health, Pitas, Sabah Malaysia; 4 Oral Health Division, Ministry of Health, Kuching, Sarawak Malaysia

**Keywords:** Dental anxiety, Children, Psychometric, Modified Child Dental Anxiety Scale faces version (MCDAS_f_)

## Abstract

**Background:**

To evaluate the psychometric properties of the faces version of the Modified Child Dental Anxiety Scale (MCDAS_f_) Malay version in 5–6 and 9–12 year-old children.

**Methods:**

The MCDAS_f_ was cross culturally adapted from English into Malay. The Malay version was tested for reliability and validity in 3 studies. In the Study 1, to determine test-retest reliability of MCDAS_f_ scale, 166 preschool children aged 5–6 years were asked to rank orders five cartoons faces depicting emotions from ‘very happy’ to ‘very sad’ faces on two separate occasions 3 weeks apart. A total of 87 other 5–6 year-old children completed the Malay-MCDAS_f_ on two separate occasions 3 weeks apart to determine test-retest reliability for Study 2. In study 3, 239 schoolchildren aged 9–12 years completed the Malay-MCDAS_f_ and the Malay-Dental Subscale of the Children Fear Survey Schedule (CFSS-DS) at the same sitting to determine the criterion and construct validity.

**Results:**

In study 1, Kendall W test showed a high degree of concordance in ranking the cartoon faces picture cards on each of the 2 occasions (time 1, W = 0.955 and time 2, W = 0.954). The Malay-MCDAS_f_ demonstrated moderate test-retest reliability (Intraclass correlation coefficient = 0.63, p <0.001) and acceptable internal consistency for all the 6 items (Cronbach’s alpha = 0.77) and 8 items (Cronbach’s alpha = 0.73). The highest MCDAS_f_ scores were observed for the items ‘injection in the gum’ and ‘tooth taken out’ for both age groups. The MCDAS_f_ significantly correlated with the CFSS-DS (Pearson r = 0.67, p < 0.001).

**Conclusions:**

These psychometric findings support for the inclusion of a cartoon faces rating scale to assess child dental anxiety and the Malay-MCDAS_f_ is a reliable and valid measure of dental anxiety in 5–12 year-old children.

## Background

Dental anxiety is a common worldwide problem affecting children as well as adults. The prevalence of dentally fearful children ranges from 3 to 55% in various populations [1-9]. Gender and age appear to be important factors linked to dental anxiety particularly common among females within the dentally anxious group in the population [10-12]. According to Hmud and Walsh [13], several factors have been shown to be related to dental anxiety including fear of pain, personal traits, traumatic dental experience during childhood and having family members or friends who are dentally anxious. The assessment of children’s dental anxiety is a concern as the unexpected behaviour of these children will have an impact on the management of this type of patients in the clinical setting [14]. In addition, it is important to develop appropriate measures for different cultures as studies had revealed that anxiety disorders are influenced by one’s culture [14-16].

There are various methods to assess dental anxiety among children. One of the methods is by using self-report measures. In self-report measures, dental anxiety score was obtained by asking the children directly about their anxiety with the assistance of rating scale of scoring. This method is usually in the form of questionnaire or interview. Most of these measures to assess child dental anxiety had been developed for the Western child population [17-20]. There is a need to examine alternative methods which may assess the dental anxiety status of young children and which provide accurate reflection of the fear they experience [19,21,22].

Over the last 15 years, several studies using cartoon faces to express children’s levels of dental anxiety as a Likert scale were used [23-28]. The Modified Child Dental Anxiety Scale faces version (MCDAS_f_) was formed by adding a cartoon faces rating scale to the original numeric form [11,20,28]. The psychometric properties of the MCDAS_f_ including the reliability, criterion and construct validity had been evaluated in different populations [11,12,15,16,22,29]. The advantage of using this self-report measure is that it is less time consuming and easy to administer. For younger children (as young as 3 years old), the questions can be read out and the children can point to the appropriate face on the scale to indicate their anxiety level. Older children (8 years and above) were able to complete the questionnaire without assistance. Additionally, the MCDAS_f_ is more versatile to be used for assessing dental anxiety over a wider age range for children from 5 to 12 years and those with limited cognitive functioning [11,12,16,22].

Limited studies on dental anxiety in children have been reported in Malaysia. A local study on refusal of dental treatment in the school dental service among 9–11 year-old schoolchildren highlighted dental anxiety as one of the reasons for refusal [30]. Another study using the Dental Fear Survey Schedule (CFSS-DS) with 10–12 year-old schoolchildren reported 15-18% of the children had high dental anxiety [7]. However, there has been no investigation of dental anxiety in preschool children and primary schoolchildren using the faces version of the MCDAS in Malaysia. It would be important to investigate if children from Malaysia could recognise the emotions expressed by cartoon faces.

The aim of this study was to evaluate the psychometric properties (reliability, criterion and construct validity) of the Malay version of the Modified Child Dental Anxiety Scale faces version (MCDAS_f_) in 5–6 and 9–12 year-old Malaysian schoolchildren. Findings from this study can be used to develop further the MCDAS_f_ as a self-report measure to assess dental anxiety in young children in Malaysia.

## Methods

### Questionnaire

In this study, the MCDAS_f_ was used as a tool to measure dental anxiety levels among 5–6 and 9–12 year-old children. This questionnaire was developed from the Modified Child Dental Anxiety Scale (MCDAS) with the addition of faces rating scale above the original numeric form to assess dental anxiety among very young and anxious children [11,20]. The MCDAS_f_ consists of questions regarding several dental procedures and the child will point to the appropriate ‘cartoon faces’ that represents their emotions or anxiety level at that time. The scale consists of eight questions about ‘going to the dentist generally’, ‘having teeth looked at’, ‘teeth being scraped and polished’, ‘injection in gum’, ‘filling’, ‘having tooth taken out’, ‘being put to sleep to have treatment’ (Dental General Anaesthesia or DGA) and ‘having a mixture of gas and air which will help you feel comfortable for treatment but cannot put you to sleep’ (Relative Analgesia or RA). Each question has five scores ranging from relaxed or not worried to very worried in an ascending order from one to five. The minimum score is 8 and the maximum score is 40.

The Malay-MCDAS_f_ was developed from its original English version. First, the English MCDAS_f_ was independently translated into Malay by a team of experts comprising a pedodontist, dental public health specialists and a psychologist. Then, a discussion on the translations of the Malay MCDAS_f_ was held by the expert group. The aim was to obtain a single Malay translation which had similar conceptual meaning with the English MCDAS_f_ using the most suitable and simple wordings in Malay. Next, the draft Malay-MCDAS_f_ was tested for face validation on 61, 5–6 year-old children (29 boys and 32 girls) from two kindergartens. The face validation testing was conducted in a classroom setting supervised by the researcher (RE). The children were asked to describe and rank order the five cartoon faces picture cards depicting emotions of very happy to very sad. This method was similar to an earlier study conducted by Humphris et al. [21]. It was found that all the children understood the meaning of the picture cards and were in agreement with each other on the rank order of the cartoon faces from very happy to very sad. Next, the MCDAS_f_ items were read out by the researcher and the children were asked to answer each question using the cartoon faces picture cards as scoring options. The time taken to answer the questions was noted.

Next, a discussion on the draft Malay-MCDAS_f_ was carried out to assess the children’s understanding on the scale’s instruction, language, content and answering technique. During the discussion, it was found that almost all of the children did not understand item 7 (related to DGA) and item 8 (related to RA). These two items will be explored further in Study 2. Back translation of the 8-item draft Malay-MCDAS_f_ was carried out by a language expert who was fluent in Malay and English language. The back translation was compared with the original MCDAS_f_ before it was finalized. Thus, the final Malay-MCDAS_f_ consisted of 8 items together with socio-demographic information, i.e. age, gender and school.

The psychometric property of the draft Malay-MCDAS_f_ was assessed by testing it on non-random groups of children in 3 separate studies:

### Study 1

#### Sample

The method employed for this study is similar to an earlier study by Humphris et al. [21]. A nonprobability sample of 181 children aged between 5 to 6 years from 5 kindergartens was invited to take part in this study. Two researchers (NAH and YA) were trained in order to standardize the interview procedure. Two sets of computer line drawings of five cartoon faces were produced to replicate the emotions depicted on the MCDAS_f_ scale. Each facial emotion was printed on a 15 cm by 10 cm index card and laminated. Every child was required to complete the task of sorting out the cards according to the scale given. The children were called individually and instructions were given by the researcher to arrange the cards accordingly from ‘very happy’ to ‘very sad’. The cards were handed to every child at random. The individual cartoon face cards were placed onto a designated Velcro board in their perceived order from ‘very happy’ to ‘very sad’. Once the child had completed the task, the cards were reshuffled to prepare for the next child. The researcher noted the order onto the data sheet for analysis. The same procedure was repeated for the same child after 3 weeks. On the second interview, only 166 children were present. Thus, the remaining fifteen children were excluded from the final analysis.

### Study 2

#### Sample

Study 2 used the faces rating scale together with the MCDAS to assess children’s dental anxiety level. A non-probability sample of another 114, 5–6 year-old children from 3 preschools completed the MCDAS_f_. The researcher read the MCDAS_f_ items and the children chose which ‘cartoon faces’ that best represented their feeling. For each MCDAS_f_ item, the response ranged from relaxed or not worried (score 1) to very worried (score 5). During the individual face to face interview, the children were briefed on the faces scale for the MCDAS_f_. The minimum and maximum score was 8 and 40 respectively. The responses were recorded on their individual forms. The MCDAS_f_ was then administered for the second time 3 weeks later.

### Study 3

For Study 3, a non-probability sample of 250, 9–12 year-old schoolchildren were invited to complete both the Malay version of MCDAS_f_ and the CFSS-DS at the same time. The aim of this study was to investigate the criterion and construct validity of the MCDAS_f_ with the CFSS-DS as the ‘gold standard’. In addition, the age and gender of the children were also recorded and tested for construct validity. Children from every class were randomly called by their teachers to the school hall in batches of 30 and the questionnaires were self-administered under standardised conditions. The researcher (RE) gave instructions prior to the survey. Two hundred and thirty-nine children completed both the MCDAS_f_ and the CFSS-DS. Eleven participants were excluded for the final analysis due to missing values.

### Statistical methods

The psychometric analysis of the draft Malay-MCDAS_f_ involved the assessment of test-retest and internal reliability, as well as face, content, criterion and construct validity.

Kendall W test for the degree of concordance was used in study 1. This test is appropriate where a set of judges is assessing the same stimuli by rank order [31]. It may also be used in the reporting of inter-test reliability [32]. Hence we calculated the degree of concordance in ranking the cartoon faces on each of the 2 occasions. It ranges from 0 (no agreement) to 1 (complete agreement) [32].

In study 2, the test-retest reliability of Malay-MCDAS_f_ overall and individual items scores between two occasions were analyzed using t-tests and intraclass correlation coefficient. The internal reliability of items in Malay-MCDAS_f_ was assessedby using Cronbach alpha coefficient.

In Study 3, the criterion validity was assessed by using the Pearson correlation coefficient and linear regression analysis to test the association between Malay-MCDAS_f_ and the CFSS-DS. For construct validity, the ANOVA test was used to test the association between gender and age with MCDAS_f_ and CFSS-DS scores, respectively. The mean (95% CI) of each item in MCDAS_f_ was also recorded.

In this study, the distribution of MCDAS_f_ (Study 1, 2 and 3) and CFSS-DS scores were found to be symmetrical. Parametric statistical tests were used. All the data analyses were carried out using SPSS version 22. The level of significance was set at p < 0.05.

### Ethical considerations

Ethical approval was obtained from the Ethics Committee, Faculty of Dentistry, University of Malaya [DF OP0809/0030(L)]. Approval to conduct the study was also obtained from the Ministry of Education, Malaysia, the State Education Director, the head teachers of all participating schools and parents of the schoolchildren involved.

## Results

### Study 1

#### Test-retest reliability of the cartoon faces picture cards

Out of 181, 5–6 year-old children (mean age = 5.53, SD ± 0.50), 166 (80 boys, 86 girls) had completed the card sort task for MCDAS_f_ rating scale on both occasions (3 weeks interval) and were included in the statistical analysis. Thus, the response rate was 92%.

Table 1 shows the value of Kendall’s W for the first and second interview. The degree of concordance in the first interview was 0.955 and the second interview was 0.954.

**Table 1:** **Degree of concordance in ranking the cartoon faces on two occasions**

Visit	N	*Kendall’s W	df	P-value
First visit	166	0.955	4	0.000
Second visit	166	0.954	4	0.000

*Kendall’s W coefficient of concordance.

### Study 2

#### Test-retest and internal reliability of the Malay-MCDAS_f_

Out of 114 children who were included in the study, 20 children were excluded as they were only present in the first interview and another 7 were excluded after data cleaning due to incongruent values of MCDAS_f_ score for the first and second interview. Thus, the final response rate was 76% (n = 87/114). The mean age was 5.60 years (SD ± 0.49) of which 48 (55.2%) were boys. For both interviews, 48.3% and 55.2% of the children did not know about the item DGA (‘being put to sleep to have treatment’). In addition, 97.7% and 100% did not know about RA (‘having a mixture of “gas and air” which will help you feel comfortable for treatment but cannot put you to sleep’).

Since this finding was in agreement with our face validation study, after further discussion with our expert group and interviewing three mothers, it was decided that both items were removed from the scale for this age group.

Table 2 shows the overall Malay-MCDAS_f_ score and the individual item mean scores between the two time intervals which were 3 weeks apart. The MCDAS_f_ mean total score was significantly higher at first administration [16.61 (95% CI: 15.68, 17.54)] than the second [14.97 (95% CI: 14.10, 15.83)] (p = 0.001). Similarly, the items ‘filling’, ‘tooth taken out’ and ‘scraped and polished’ scored significantly higher at first administration than the second, respectively (p < 0.05). The other items i.e. ‘dentist generally’, ‘teeth looked at’, and ‘injection in the gum’ showed no significant differences between the two time intervals (P > 0.05). For both assessments, the highest MCDAS_f_ scores were observed for the items ‘injection in the gum’ and ‘tooth taken out’, respectively. In terms of test-retest reliability, the intraclass correlation coefficients demonstrated good correlation with scores ranging from 0.65 to 0.77 (p < 0.001) for the individual items of the MCDAS_f_ between the first and second assessments. The intraclass correlation coefficient for the mean overall MCDAS_f_ score was 0.63 (p < 0.001) between the first and second assessments. In terms of internal reliability of the Malay-MCDAS_f_, the corrected item-total correlation values were all positive and above 0.3, i.e. between 0.43 to 0.67. The Cronbach’s alpha was 0.77 and the value did not increase if any of the 6 items were deleted (Table 3).

**Table 2:** **Overall and Individual item mean scores of Malay-MCDAS**_**f**_ **between two time intervals for 5–6 year-old children**

N = 87	First visit	Second visit	t value	P-value
	Mean (95% CI)	Mean (95% CI)		
Mean MCDAS_f_ Score	16.61 (15.68, 17.54)	14.97 (14.10, 15.83)	3.50	0.001
Dentist generally	1.76 (1.54, 1.98)	1.57 (1.39, 1.76)	−1.43	0.155
Teeth looked at	1.91 (1.71, 2.11)	1.91 (1.72, 2.09)	0.00	1.000
Scraped and polished	2.28 (2.04, 2.51)	1.86 (1.65, 2.07)	−2.60	0.011
Injection in the gum	3.66 (3.43, 3.88)	3.77 (3.49, 4.05)	0.88	0.380
Filling	2.95 (2.70, 3.21)	2.18 (1.93, 2.44)	−4.74	0.001
Tooth taken out	4.06 (3.84, 4.28)	3.67 (3.38, 3.95)	−2.79	0.006

Paired *t* Test.

Two items, ‘dental general anaesthesia’ and ‘relative analgesia’ items were omitted for this age group.

**Table 3:** **Reliability analysis: corrected item-total correlation of the 6 items of the Malay-MCDAS**_**f**_ **and Cronbach’s Alpha coefficients for 5–6 year-old children**

	Corrected item-total correlation	Cronbach’s alpha if item deleted
Dentist generally	.429	.76
Teeth looked at	.483	.75
Scraped and polished	.526	.74
Injection in the gum	.583	.72
Filling	.446	.76
Tooth taken out	.671	.70
Alpha value		.77
Standardised items alpha		.78

Two items, ‘dental general anaesthesia’ and ‘relative analgesia’ items were omitted for this age group.

### Study 3

#### Criterion and construct validity of Malay-MCDAS_f_

Out of 250, 9–12 year-old schoolchildren who were invited to complete both the Malay-MCDAS_f_ and the CFSS-DS, 11 participants were excluded due to missing values. Two hundred and thirty-nine children completed both the MCDAS_f_ and the CFSS-DS. Another 9 participants did not know or did not respond to the 2 items on DGA and RA. Thus they were excluded from the final analysis giving a final response rate of 92% (n = 230/250) and the 8-item Malay-MCDAS_f_ was used for this age group. Majority were Malays (72.2%), followed by Indians (23%), Chinese (3%) and others (1.7%). There were slightly more boys (n = 121, 52.6%) and their mean age was 9.87 years (SD ± 0.77).

The mean overall score for the MCDAS_f_ was 21.77 (95% CI: 21.01, 22.53), with a range of scores from 8 to 40. The mean overall score for the CFSS-DS was 37.57 (95% CI: 36.09, 39.05), with a range of scores from 15 to 71. In this age group, the corrected item-total correlation values were all positive and above 0.3, i.e. between 0.31 to 0.54 for MCDAS_f_ (Table 4). Similarly, the corrected item-total correlation values for CFSS-DS were also all positive and above 0.3, i.e. between 0.31 to 0.65. The Cronbach’s alpha coefficients of MCDAS_f_ and CFSS-DS were 0.73 and 0.87, respectively.

**Table 4:** **Reliability analysis: corrected item-total correlation of the 8 items of the Malay-MCDAS**_**f**_ **and Cronbach’s Alpha coefficients for 9–12 year-old children**

	Mean (SD)	Corrected item-total correlation	Cronbach’s alpha if item deleted
Dentist generally	1.96 (0.98)	.310	.72
Teeth looked at	2.23 (1.15)	.539	.68
Scraped and polished	2.60 (1.28)	.473	.69
Injection in the gum	3.90 (1.21)	.435	.70
Filling	2.82 (1.36)	.499	.68
Tooth taken out	3.63 (1.34)	.431	.70
DGA*	2.15 (1.26)	.333	.72
RA*	2.48 (1.40)	.353	.72
Alpha value			.73
Standardised items alpha			.73

*DGA - Dental General Anaesthesia.

*RA – Relative Analgesia.

For criterion validity testing, the mean overall scores for the MCDAS_f_ and the CFSS-DS were significantly correlated (Pearson r =0.67, p < 0.001), where 45% of the variance in CFSS-DS was explained by MCDAS_f_ (Table 5).

**Table 5:** **Summary statistics of linear regression analysis for MCDAS**_**f**_ **and CFSS-DS**

Dependent	R	R square	Std. error	df	F	Sig.
CFSS-DS	0.672	0.452	4.366	1	187.698	0.000

Predictors: (Constant), MCDAS_f_ score.

Dependent variable: CFSS-DS score.

For construct validity, the variance of dental anxiety as assessed by MCDAS_f_ and CFSS-DS was analysed across gender and age (Tables 6 and 7). Girls had significantly higher mean scores for dental anxiety compared to boys for both the MCDAS_f_ and CFSS-DS (p < 0.001). There were no significant differences in mean scores between the three age groups for both the MCDAS_f_ and CFSS-DS (p > 0.05).

**Table 6:** **Association between MCDAS**_**f**_ **scores with demographic characteristics of the school children**

Demographic variable	N (%)	Mean MCDAS_f_	95% CI	p-value
Gender				
Boys	121 (52.6)	20.45	19.42, 21.48	0.000
Girls	109 (47.4)	23.23	22.14, 24.32	
Age (years)				
9	79 (34.3)	21.14	19.78, 22.50	
10	107 (46.5)	21.93	20.84, 23.02	0.436
*11-12	44 (19.2)	22.50	20.68, 24.32	
Overall Mean MCDAS_f_ score	230	21.77	21.01, 22.53	

*5, 12-year-old children were included in the 11-year-old group as they were in the same grade.

**Table 7:** **Association between CFSS-DS scores with demographic characteristics of the school children**

Demographic variable	N (%)	Mean CFSS-DS	95% CI	p-value
Gender				
Boys	121 (52.6)	35.26	33.34, 37.18	0.001
Girls	109 (47.4)	40.14	37.92, 42.35	
Age (years)				
9	79 (34.3)	37.80	35.27, 40.32	
10	107 (46.5)	37.97	35.73, 40.21	0.666
*11-12	44 (19.2)	36.18	32.84, 39.53	
Overall Mean CFSS-DS score	230	37.57	36.09, 39.05	

*5, 12-year-old children were included in the 11-year-old group as they were in the same grade.

## Discussion

In this study, the development of the Malay-MCDAS_f_ followed a recommended method [33] and was closely related to other similar studies on the index validation [11,23,24,29] . The forward and back translations of the 8-item MCDAS_f_ were done by local experts in dental public health, pediatric dentistry, child psychologist and Malay and English languages. The experts unanimously agreed that the Malay-MCDAS_f_ and its original English version had similar subject content and meanings of its items. The final back translation of Malay-MCDAS_f_ into its English version contained many similar words with the original MCDAS_f_. The slight differences in the wordings between the two were due to cultural variations and children’s preferences in the choice of words during pretest of the Malay MCDAS_f_. In general, the study findings suggest that the Malay-MCDAS_f_ has high potential to measure dental anxiety among Malaysian preschool and schoolchildren. Its linguistic validation, i.e. face and content validity and psychometric validation, i.e. internal reliability, test-retest reliability, criterion and construct validity had been tested and verified by experts and statistical analyses.

In Malaysia, children start primary school at the age of 7 until 12 years, and at 5 to 6 years they are in preschool. The oral healthcare programme for preschool children launched in 1984, covers 5–6 year-old children attending kindergartens and pre-schools. This programme focuses on a friendly, non-invasive approach whereby dental nurses introduced dentistry to children via promotional and preventive initiatives and visit the children twice a year. These include tooth brushing sessions, puppet shows, role-play and other fun activities. The Atraumatic Restorative Technique (ART) is adopted to provide necessary restorative care for the children [34]. The three studies were conducted simultaneously to evaluate the overall psychometric properties of the Malay-MCDAS_f_ to validate its use by both preschool and primary schoolchildren in Malaysia.

At the face validation stage, it was found that the majority of the 5–6 year-old children were not able to distinguish between DGA and RA with the majority of children stated they were unafraid. When the children were asked about these procedures it became apparent that they did not know about DGA or RA [22]. This finding was not surprising as general anesthesia is used selectively for dental extractions in Malaysia [35] and RA is rarely used for paediatric dental patients in Malaysia. Furthermore, evidence had shown that the most effective drug or method of sedation used for anxious children are still debatable [36]. Additionally, Study 2 also showed that majority did not know about DGA and almost all were not aware of RA. Therefore it was decided to omit these two items from the Malay-MCDAS_f_ for the 5–6 year-old children due to their lack of experience and understanding of both types of treatments. However, these two items were used for the 9–12 year-old children. Christophorou et al. [12] and Javadinejad et al. [16] had also employed the 8-item version of the MCDAS for 8–12 year-old primary schoolchildren.

The results from Study 1 indicated that preschool children in Malaysia possessed the right levels of cognitive and affective abilities to discriminate between happy and sad faces on the cartoon faces rating scale. The degree of concordance was high and almost similar for the first as well as the second administration of the play board of cartoon faces. The Kendall’s W coefficient of concordance value showed nearly perfect ranking from very happy, happy, neutral, sad, and very sad emotions. This finding was in resemblance with findings from other similar studies which reported consistent rank ordering employing cartoon faces (very happy-happy-sad-very sad) or using the Facial Image Scale in the assessment of dental anxiety in young children [23-25].

In Study 2, in terms of its reliability test, the corrected item-total correlation values for all the 6 items were well above 0.2, indicating all the Malay-MCDAS_f_ items correlated well with the sum score. This indicated the 6 items were stable to form an index and had high internal reliability [32]. Also, the Cronbach’s alpha value was 0.77 indicating the 6 item Malay-MCDAS_f_ was reliable to measure dental anxiety among 5–6 year-old preschool children in Malaysian setting. If any of the items were removed, the value of Cronbach’s alpha did not increase. This indicated no item should be removed to further improve the scale reliability. As for the test-retest reliability of the Malay-MCDAS_f_, the relatively high intraclass correlation coefficient values of individual items and mean scores when the scale was fielded at two different times indicated the Malay-MCDAS_f_ was reliable in producing consistent outcomes.

The differences in the mean scores for the six items of the Malay-MCDAS_f_ showed that children were able to discriminate between more invasive treatment procedures such as ‘tooth taken out, ‘injection’, ‘filling’ and ‘scraped and polished’ which had higher mean scores compared to less invasive treatment such as ‘having teeth looked at’ or ‘dentist generally’. The slightly lower mean scores obtained for the second test could be explained by a practice effect since the children were already familiar with the items. However, for ‘injection in the gums’ the score for the second test was slightly higher than the first test but it was not statistically significant. Howard and Freeman [11] similarly cited that the item ‘injection in the gums’ as the greatest level of dental anxiety. Interestingly, the 8 item Malay-MCDAS_f_ for the 9–12 year-old children also highlighted similar anxiety provoking stimulus. The item scores for DGA and RA were quite similar to the non-invasive items of the MCDAS_f._ possibly due to non-exposure to these procedures.

Howard and Freeman [11] showed that a two-factor structure namely ‘examination’ and ‘treatment’ existed for the MCDAS_f_ in their sample. However, Zhang et al. [29] confirmed that the Chinese version of the MCDAS_f_ scale consisted of a single unidimensional construct (written in Chinese). Similarly, Christophorou et al. [12] also confirmed a clear unidimensional structure for the Greek version of MCDAS. Hence it would be preferable to test the Malay-MCDAS_f_ on a larger sample size so that a confirmatory factor analytic approach and structural equation modelling could be applied. This would help to confirm if a two-factor structure for the Malay version of the MCDAS_f_ existed in this child population.

In Study 3, the Malay-MCDAS_f_ was tested against the Malay-CFSS-DS which is the ‘gold standard’ among 9–12 year-old children to measure dental anxiety [11,22,37,38]. Although the internal consistency of MCDAS_f_ was lower being 0.73 as compared to 0.86 for CFSS-DS, it was still within the acceptable range for Cronbach’s alpha [39]. A possible explanation for this difference could be due to the fact that the MCDAS_f_ only contained 8 items compared to 15 items of CFSS-DS used in this study. Nevertheless, there was a significant correlation between the mean overall scores for the MCDAS_f_ and the CFSS-DS where 45% of the variance in CFSS-DS was explained by MCDAS_f_. Both these findings indicated that MCDAS_f_ has good internal consistency and good criterion validity in relation to the CFSS-DS.

In terms of the construct validity, the variance of dental anxiety as assessed by MCDAS_f_ and CFSS-DS demonstrated similar findings that girls had significantly higher mean scores for dental anxiety compared to boys. With regard to age groups, both the MCDAS_f_ and CFSS-DS showed no significant difference in mean scores between the three age groups. These findings indicated that the Malay-MCDAS_f_ had similar construct validity with the Malay-CFSS-DS in the Malaysian setting. Thus, it could be said that the Malay-MCDAS_f_ was equally valid to assess dental anxiety among school children in Malaysia similar to the ‘gold standard’, CFSS-DS (Malay version).

One of the limitations of this study was the sample. All three studies used urban children with varying socioeconomic background and adequate gender distribution. It is recommended that future study to further validate the Malay-MCDAS_f_ is conducted on a larger sample of rural children and other ethnic groups. Further longitudinal study on the same population is also recommended to assess dental anxiety over time.

Previous studies had established that children and adolescents with high caries experience exhibited high dental anxiety [11,40,41]. Another limitation of this study is that MCDAS_f_ was not tested against any clinical measure of oral health or in a clinical situation between anxious and non-anxious children. Further studies should look into these areas and establish suitable cut off level for dental anxiety in this population. Despite these limitations, the 6-item and 8-item Malay-MCDAS_f_ has been shown to be valid and reliable to be used as a screening tool across a wider age range in children survey as well as prior to dental procedures in the clinic.

## Conclusions

The psychometric properties of the Malay-MCDAS_f_ provided empirical evidence to support for the inclusion of a cartoon faces rating scheme in the scale to assess child dental anxiety in children. The 6-item Malay-MCDAS_f_ is recommended for assessing dental anxiety for 5–6 year-old and younger children whereas the 8-item Malay-MCDAS_f_ can be used for older children. Both versions had been verified to be valid and reliable to measure dental anxiety in 5–12 year-old children.

## Abbreviations

ANOVA: Analysis of variance
ART: Atraumatic restorative technique
DGA: Dental general anaesthesia
CFSS-DS: Dental subscale of the children fear survey schedule
MCDAS: Modified child dental anxiety scale
MCDAS_f_: Modified child dental anxiety scale faces version
RA: Relative analgesia
